# Variations in Abdominal Aortic Aneurysm Care: A Report From the International Consortium of Vascular Registries

**DOI:** 10.1161/CIRCULATIONAHA.116.024870

**Published:** 2016-12-12

**Authors:** Adam W. Beck, Art Sedrakyan, Jialin Mao, Maarit Venermo, Rumi Faizer, Sebastian Debus, Christian-Alexander Behrendt, Salvatore Scali, Martin Altreuther, Marc Schermerhorn, Barry Beiles, Zoltan Szeberin, Nikolaj Eldrup, Gudmundur Danielsson, Ian Thomson, Pius Wigger, Martin Björck, Jack L. Cronenwett, Kevin Mani

**Affiliations:** From Division of Vascular Surgery and Endovascular Therapy, University of Alabama, Birmingham (A.W.B.); Healthcare Policy and Research, Weill Cornell Medical College, New York, NY (A.S., J.M.); Department of Vascular Surgery, Helsinki University Hospital, Helsinki, Finland (M.V.); Division of Vascular Surgery, University of Minnesota, Minneapolis (R.F.); Department of Vascular Medicine, University Heart Center Hamburg-Eppendorf, Hamburg, Germany (S.D., C.-A.B.); Division of Vascular Surgery and Endovascular Therapy, University of Florida, Gainesville (S.S.); Department of Vascular Surgery, St. Olavs Hospital, Trondheim, Norway (M.A.); Division of Vascular and Endovascular Surgery, Beth Israel Deaconess Medical Center, Boston, MA (M.S.); Australian and New Zealand Society for Vascular Surgery, East Melbourne, Australia (B.B.); Department of Vascular Surgery, Semmelweis University, Budapest, Hungary (Z.S.); Department of Cardio-Thoracic and Vascular Surgery, Aarhus University Hospital, Aarhus, Denmark (N.E.); National University Hospital of Iceland, Department of Surgery, Reykjavík, Iceland (G.D.); Department of Vascular Surgery, Dunedin School of Medicine, Dunedin Hospital, Dunedin, New Zealand (I.T.);Department of Cardiovascular Surgery, Kantonsspital Winterthur, Winterthur, Switzerland (P.W.); Department of Surgical Sciences, Vascular Surgery, Uppsala University, Uppsala, Sweden (M.B., K.M.); and Section of Vascular Surgery, Dartmouth-Hitchcock Medical Center, Lebanon, NH (J.L.C.).

**Keywords:** aortic aneurysm, abdominal, aortic rupture, practice patterns, physicians’, quality improvement, registries

## Abstract

**Background:**

This project by the ICVR (International Consortium of Vascular Registries), a collaboration of 11 vascular surgical quality registries, was designed to evaluate international variation in the contemporary management of abdominal aortic aneurysm (AAA) with relation to recommended treatment guidelines from the Society for Vascular Surgery and the European Society for Vascular Surgery.

**Methods:**

Registry data for open and endovascular AAA repair (EVAR) during 2010 to 2013 were collected from 11 countries. Variations in patient selection and treatment were compared across countries and across centers within countries.

**Results:**

Among 51 153 patients, 86% were treated for intact AAA (iAAA) and 14% for ruptured AAA. Women constituted 18% of the entire cohort (range, 12% in Switzerland–21% in the United States; *P*<0.01). Intact AAAs were repaired at diameters smaller than recommended by guidelines in 31% of men (<5.5 cm; range, 6% in Iceland–41% in Germany; *P*<0.01) and 12% of women with iAAA (<5 cm; range, 0% in Iceland–16% in the United States; *P*<0.01). Overall, use of EVAR for iAAA varied from 28% in Hungary to 79% in the United States (*P*<0.01) and for ruptured AAA from 5% in Denmark to 52% in the United States (*P*<0.01). In addition to the between-country variations, significant variations were present between centers in each country in terms of EVAR use and rate of small AAA repair. Countries that more frequently treated small AAAs tended to use EVAR more frequently (trend: correlation coefficient, 0.51; *P*=0.14). Octogenarians made up 23% of all patients, ranging from 12% in Hungary to 29% in Australia (*P*<0.01). In countries with a fee-for-service reimbursement system (Australia, Germany, Switzerland, and the United States), the proportions of small AAA (33%) and octogenarians undergoing iAAA repair (25%) were higher compared with countries with a population-based reimbursement model (small AAA repair, 16%; octogenarians, 18%; *P*<0.01). In general, center-level variation within countries in the management of AAA was as important as variation between countries.

**Conclusions:**

Despite homogeneous guidelines from professional societies, significant variation exists in the management of AAA, most notably for iAAA diameter at repair, use of EVAR, and the treatment of elderly patients. ICVR provides an opportunity to study treatment variation across countries and to encourage optimal practice by sharing these results.

## Abstract 2

Supplemental Digital Content is available in the text.

**Editorial, see p 1959**

Abdominal aortic aneurysm (AAA) is a common disease in the Western population, with a prevalence of 2% to 5% in men ≥65 years of age,^1–3^ and is a major cause of death as a result of rupture.^4^ Treatment consists of an open or endovascular aortic repair (EVAR).

Many aspects of treatment, including size threshold for intervention,^5–8^ the benefits of EVAR,^9–12^ and AAA screening in high-risk cohorts,^1,13–15^ have been studied extensively. Using the available data, both the Society for Vascular Surgery (SVS) and the European Society for Vascular Surgery (ESVS) have established guidelines for the management of AAA.^16,17^ The aim of these guidelines is to settle uncertainties in management and to identify best practices. However, treatment choices may be driven not only by clinical and medical science but also by cultural differences, economic incentives, access to technology, physician skill set, and patient/physician preference.^18^ Historically, significant variation in the management of AAA has been noted between some countries, but wider international variation related to society guidelines has not been assessed.^19–22^

Here, we assessed differences in contemporary practice for 11 countries across 3 continents participating in the ICVR (International Consortium of Vascular Registries),^23^ which is a collaboration of national quality improvement registries.

## Methods

Unidentifiable data from registries in 11 countries were submitted to the Medical Device Epidemiology Network Analytic Center at Weil Cornell University for analysis. The Table demonstrates the countries involved and provides background data for each registry. Because of the nature of their registries, procedures performed during the studied time period were covered on the national level in Australia, Denmark, Hungary, Iceland, New Zealand, Norway, and Sweden. The Finnish registry captured AAA repairs in 3 hospital regions (Helsinki, central Finland, and South Karelia), and the Swiss registry captured procedures performed within the Swiss public healthcare sector. Data from Germany included AAA repairs from ≈100 centers participating in the German vascular registry. In the United States, 150 centers participating in the SVS Vascular Quality Initiative EVAR and open AAA registries were used in the analysis. These centers represent an estimated 15% of the aneurysm repairs performed in the United States over the time studied and essentially 100% of cases performed at each participating institution. Data for each Vascular Quality Initiative center are audited against claims data to ensure consecutive case entry.

**Table.**
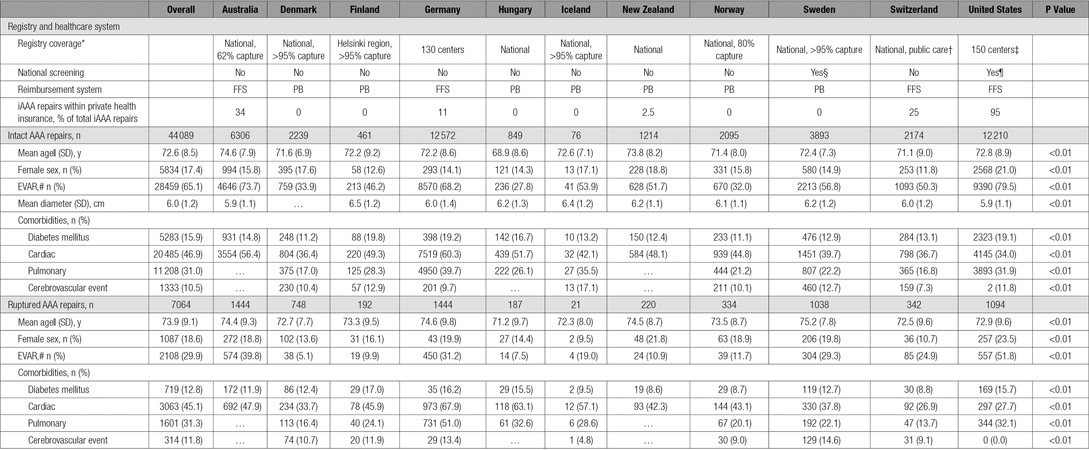
National and Patient Characteristics for AAA Repair

All primary AAA repairs (intact [iAAA] and ruptured [rAAA]) collected between January 2010 and December 2013 were included (Figures I and II in the online-only Data Supplement). For Vascular Quality Initiative centers specifically, when the use of EVAR versus open AAA repair was examined, only centers participating in both the EVAR and open repair registries were included for that portion of the analysis (n=121). All analyses focusing on AAA size were limited to intact repairs for which AAA size was available. Cases with a registered AAA diameter of <4 cm were excluded because these procedures are likely to have been performed for indications other than degenerative atherosclerotic aortic aneurysms (eg, iliac aneurysms, penetrating ulcer, dissection).

### Statistical Analysis

The study was planned and performed in accordance with the STROBE (Strengthening the Reporting of Observational Studies in Epidemiology) guidelines for observational studies.^24^ Demographics, comorbidities (diabetes mellitus, cardiac, pulmonary, and renal and cerebrovascular disease), and operative data, including year of repair, indication (rupture/intact, AAA diameter), and technique (open/EVAR), were evaluated. The Norwegian registry provided age in 5-year groups rather than exact age, so estimates were derived from the median for each 5-year age group. Comorbidity definitions/severity varied slightly across registries (Figure III in the online-only Data Supplement). Of note, both the SVS and the ESVS recommend a diameter threshold of ≥5.5 cm for elective repair in men, but they differ slightly in the recommendation for women (SVS, ≥5.0 cm; ESVS, ≥5.2 cm). For the purposes of this analysis, small iAAA repair is defined as <5.5-cm diameter in men and <5-cm diameter in women.

Patient characteristics were presented as mean and SD for age and aneurysm diameter and as percentages for proportions. One-way ANOVA and χ^2^ tests were used for assessment of inference for continuous variables (age and diameter) and proportions, respectively. Variations in patient characteristics and use of EVAR were assessed on both the country and center levels. Proportions and their 95% Wald confidence intervals were obtained. Center-level data were available for a subset of the patients in the German registry and not at all for the vascular registries in Norway and New Zealand. For center-level correlation assessment, procedures performed in Germany, Norway, and New Zealand were not included. To assess variations in practice on the basis of case volume, center volume variations were assessed by dividing centers in quartiles on the basis of averaged annual overall number of AAA repairs.

Covariations between various parameters (proportion of EVAR for iAAA, proportion of EVAR in rAAA, and proportion of small AAA repair) on both the country and center levels were assessed with the Pearson correlation test. Analysis of trend was performed within centers that enrolled in registries for all 4 years. A Cochrane-Armitage test for trend was used to assess the trend of proportions of ruptured aneurysm repair and small aneurysm repair overall and within each country. Missing values were excluded from analysis. Countries were further evaluated on the basis of their healthcare economic model. To compare differences in small aneurysm repair and octogenarians undergoing intact aneurysm repair between countries with different healthcare economic models, a generalized linear mixed model accounting for center and country clustering was used, adjusting for procedure year. To adjust for multiple comparisons, a value of *P*<0.01 was regarded as significant. All analyses were performed with SAS version 9.3 (SAS Institute Inc, Cary, NC).

Ethics approval for the collection and analysis of vascular registry data was obtained on the basis of national regulations for each registry for this international collaborative project.

## Results

A total of 51 153 patients were identified, 44 089 with iAAA (86%) and 7064 with rAAA (14%). Ruptured AAA rates (as a percentage of all treated AAA) ranged from 8% in the United States to 29% in Finland (*P*<0.001). Overall, there was a trend toward lower rate of rAAA repair over time (19.0% in 2010 to 17.6% in 2013; *P*=0.16).

There were important demographic differences for a number of variables (Table). Women represented 18% of all treated AAAs (range, 12% in Switzerland–21% in the United States; *P*<0.01). Mean age was 72.5 years for men and 74.7 years for women at treatment (range in men, 68.8 years in Hungary–74.1 years in Australia, *P*<0.01; range in women, 72.1 years in Hungary–76.6 years in Australia, *P*<0.01). Octogenarians represented 23% of all patients (men, 22% [range, 12% in Hungary–27% in Australia]; women, 30% [range, 15% in Hungary–41% in Australia]; *P*<0.01). The lowest-volume centers (first quartile) performed <30 AAA repairs per year, whereas the highest-volume centers (fourth quartile) performed ≥95 repairs per year. The proportion of iAAA repairs performed at low-volume centers varied between countries from 20% in Sweden and the United States to 46% in Australia (*P*<0.001).

### Aneurysm Diameter at Repair

**Figure 1.**
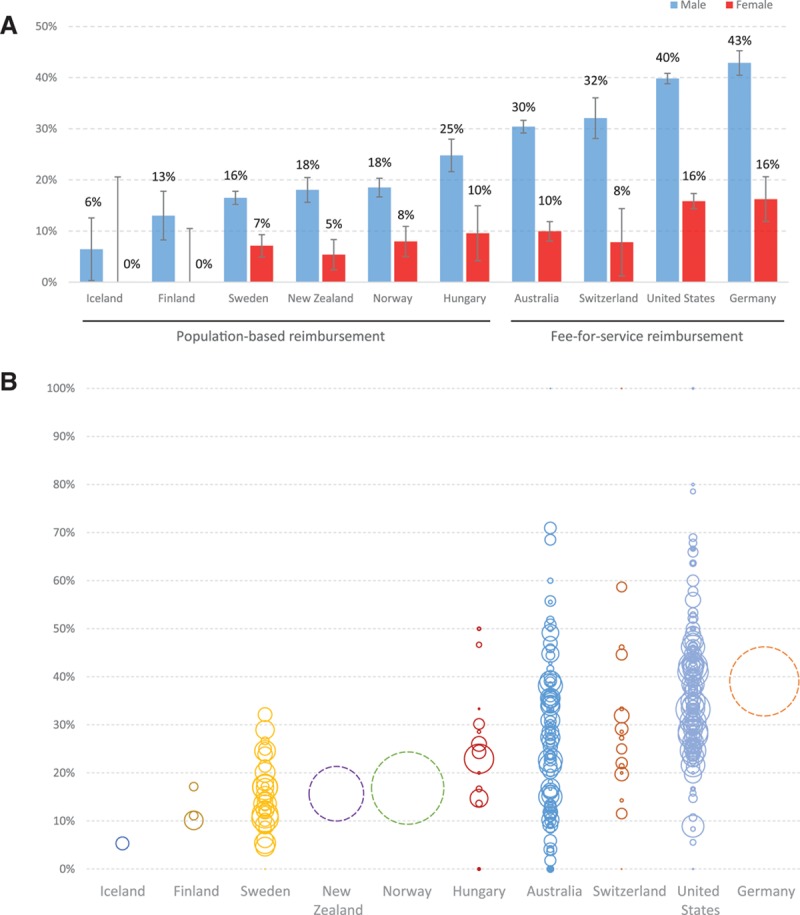
**Variations in elective management of abdominal aortic aneurysm (AAA). A**, Proportion of patients undergoing intact AAA repair at a diameter <5.5 cm for men (blue bars) and <5.0 cm in women (red bars) in each country. Both *P*<0.01 for difference between countries. **B**, Center-level analysis of proportion of patients undergoing intact AAA repair at an aneurysm diameter <5.5 cm for men and <5.0 cm in women. Each circle represents a center, and the size of the circle represents the volume of AAA repair at that center. Center-level data were not available for Germany, Norway, and New Zealand, which are represented by dotted circles.

Overall, 28% of iAAA repairs were for small AAAs (from 5% in Iceland–39% in Germany). The rate of small AAA was higher for those undergoing EVAR (31%) compared with open repair (21%; *P*<0.01). Most patients undergoing repair for small AAA had a diameter close to (within 0.5 cm of) the recommended threshold (men, 73%; women, 74%). There was wide international variation in rate of small iAAA repair. On average, 31% of iAAA repairs were performed at a diameter <5.5 cm in men (range, 6% in Iceland–43% Germany; *P*<0.01) and 12% at <5-cm diameter in women (range, 0% in Iceland and Finland–16% in the United States and Germany; *P*<0.01; Figure 1A). As a result of the uncertainties in the ESVS and SVS recommendation for iAAA repair threshold for women, a separate analysis was performed to assess the proportion of women undergoing repair for iAAA <5.5 and <5.2 cm in each country (Figure IV in the online-only Data Supplement). This analysis confirmed all the previously mentioned variations between countries. In addition to the between-country variation in proportion of small AAAs repaired, there was significant variation between centers in each country in terms of the proportion of intact AAA repairs performed for small AAA (Figure 1B). Overall, in low-volume centers, small AAA constituted 29% of the iAAA repairs compared with 28% in high-volume centers (*P*=0.08). An assessment of trends in the percentage of small iAAA repair over the study period did not show a significant trend overall or in specific countries (data not shown).

### Operative Technique

**Figure 2.**
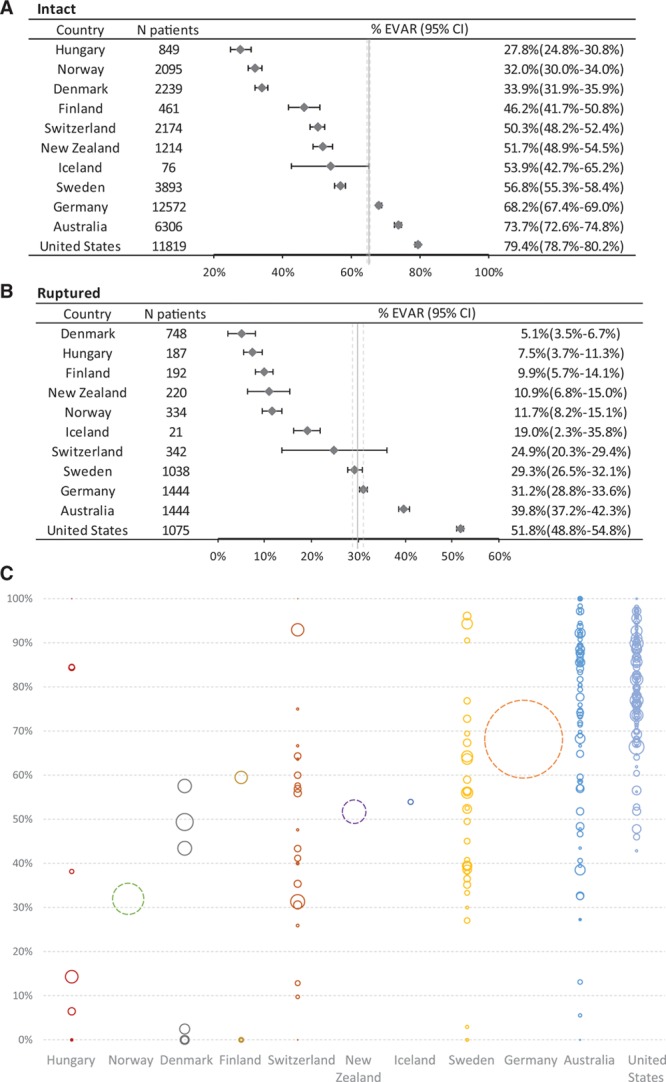
**Variations in modality of repair (open vs endovascular aortic repair [EVAR]).** Proportion of patients undergoing EVAR for intact (**A**) and ruptured (**B**) abdominal aortic aneurysm (AAA). **C**, Presentation of center-level variation within each country for the proportion of patients undergoing EVAR for intact AAA repair. Each circle represents a center, and the size of the circle represents the volume of AAA repair at that center. Center-level data were not available for Germany, Norway, and New Zealand, which are represented by dotted circles.

**Figure 3.**
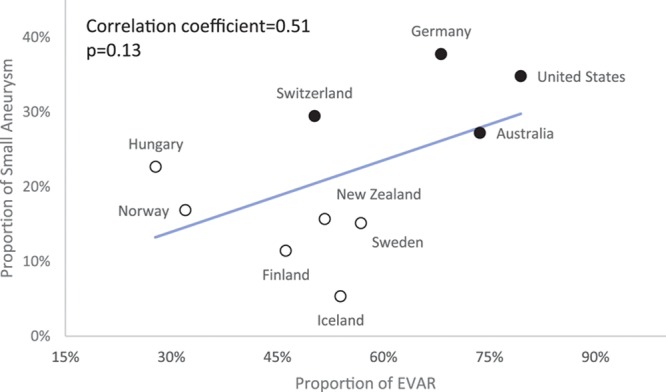
**Aneurysm diameter related to endovascular aortic repair (EVAR) and healthcare reimbursement model.** Correlation of the proportion of small intact aneurysm repair per country and use of EVAR for intact abdominal aortic aneurysm repair. White dots represent countries with a population-based reimbursement system, and black dots represent countries with fee-for-service reimbursement for surgical care. CI indicates confidence interval.

Overall, EVAR was used in 65% of iAAA repairs and 30% of rAAA repairs. EVAR was used in 65% of iAAA repairs in men and less often in women (60%; *P*<0.01). There were marked differences in the use of EVAR for iAAA repair between the countries with the lowest (Hungary, 28%) and highest (United States, 79%) use (*P*<0.01). Fewer than 40% of iAAA repairs in Hungary, Norway, and Denmark were performed as EVAR, whereas >60% of iAAA repairs in Germany, Australia, and the United States were EVAR. EVAR rates for iAAA in other countries varied between 40% and 60% (Figure 2A). There was a similar wide variation across countries in the use of EVAR for rAAA (Figure 2B). There was a correlation between EVAR use in the iAAA and rAAA setting on the national level (correlation coefficient, 0.92; *P*<0.01) and on the center level (correlation coefficient, 0.60; *P*<0.01). There was a trend toward a correlation between the proportion of patients treated for small AAA and the use of EVAR for iAAA repair on the country level (correlation coefficient, 0.51; *P*=0.13) and center level (correlation coefficient, 0.27; *P*<0.01; Figure 3).The results of the correlation analyses were consistent when performed across all years and when individual year data were analyzed. Analysis of operative technique at the center level showed a significant variation in use of EVAR in AAA repair between centers within each country, for example, from 0% to 100% in Australia and 0% to 96% in Sweden (Figure 2C).

### Healthcare Reimbursement Models

**Figure 4.**
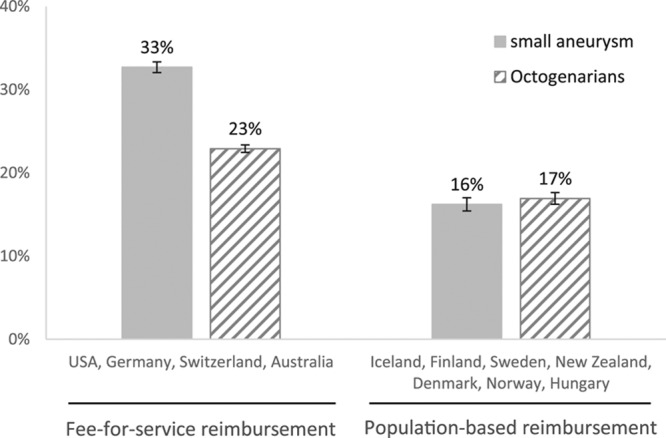
**Healthcare reimbursement related to management of small aneurysms and elderly patients.** Comparison of countries with a fee-for-service reimbursement system and those with a population-based reimbursement system in surgical care: proportions of patients with small aneurysm and proportions of octogenarians undergoing intact abdominal aortic aneurysm repair. Both *P*<0.01.

Australia, Germany, Switzerland, and the United States represent countries where the healthcare reimbursement model is based, at least in part, on fee for service (Table). All other countries in the ICVR provide AAA treatment primarily within a population-based reimbursement model. The proportion of small AAAs and the proportion of octogenarians undergoing iAAA repair were higher in fee-for-service countries compared with population-based reimbursement countries (Figure 4). In a comparison using hierarchical analysis accounting for center- and country-level clustering and adjusting for procedure year, fee-for-service countries had significantly more small AAA repair (odds ratio, 2.25; 95% confidence interval, 1.35–3.78; *P*<0.01) and slightly more octogenarians undergoing iAAA repair (odds ratio, 1.49; 95% confidence interval, 0.88–2.53; *P*=0.12) than countries with population-based reimbursement.

### Threshold Diameter for Elective Repair Versus Rupture Rate

As noted, the proportion of repairs for rAAA varied significantly between countries. Because more frequent repair of small iAAA could bias this outcome, variation in the proportion of iAAA and rAAA repairs only in patients with ≥5.5-cm AAAs was also evaluated. This did not alter the wide variation seen between countries (rAAA as a proportion of all AAA repair ≥5.5 cm, 16%; range, 11% in United States–40% in Finland; *P*<0.01).

## Discussion

This is the first analysis from a multinational collaboration of vascular quality improvement organizations from sites in Australia, Europe, New Zealand, and the United States. This analysis demonstrated significant variation across countries in the rate of small iAAA treatment, the preferred surgical approach (EVAR versus open), the proportion of elderly patients undergoing repair, and the proportion of repairs performed for rupture. Variations existed both between countries and between centers within each country. The differences in indication for iAAA repair on the basis of the healthcare reimbursement model suggested differential management on the basis of positive or negative financial incentives. The analyses point toward the challenges in providing uniform health care to patients in different countries and centers despite existing societal guidelines.

Both the SVS and ESVS recommend a size threshold of 5.5 cm for iAAA repair in men. The recommendation is less uniform in women with 5.0 cm by the SVS^16^ and 5.2 cm by the ESVS.^17^ Despite this, there was significant variation both within and across Australia, Europe, New Zealand, and the United States in the proportion of iAAAs being treated below the recommended diameter threshold. The current SVS and ESVS guidelines are based in part on 2 randomized studies in the endovascular era, 1 American study (PIVOTAL study [Positive Impact of Endovascular Options for Treating Aneurysms Early])^7^ and 1 European study (CAESAR study [Comparison of Surveillance Versus Aortic Endografting for Small Aneurysm Repair]),^8^ demonstrating that there is no survival benefit with repair of small AAA. This is due to the fact that rupture risk of AAAs <5.5 cm in size is <1%/y on the basis of randomized trials.^5–8^ In a previous registry-based analysis, the perioperative mortality after intact AAA repair for aneurysms <5.5 cm was 0.7% for EVAR and 2.7% for open repair.^20^ These results were in line with the randomized trials^7,8^ and indicate that repair of small AAA is unlikely to result in an overall survival benefit, even when performed with EVAR. With regard to open repair, 2 large, randomized trials did not show any benefit from repair of AAA <5.5 cm in size over 9 and 12 years of follow-up.^5,6^

The majority of repairs for small iAAA were in patients with a diameter within the 0.5-cm range below the treatment threshold (5–5.4 cm in men and 4.5–4.9 mm in women), and small AAA repair was more prevalent with EVAR. The clinical rationale for repair at a small diameter is that more than half of the patients progress and meet the threshold for repair within 2 years of follow-up.^8^ However, the operative morbidity and mortality of iAAA repair cannot be neglected. It is important to note that patients with AAA have a lower life expectancy than age-matched cohorts because of disease in other vascular beds, advanced age, malignancy, and pulmonary disease,^7,8^ and repair of a small AAA with a low risk of rupture is unlikely to improve the overall survival. Although we do not have complete data on the prior AAA expansion rate or symptomatic status as an indication for intact AAA repair, these effects should be similar across countries and are unlikely to explain the observed wide variation in practice.

The potential effects of different reimbursement models are provocative. The current data set does not allow differentiation of whether repair among patients with small AAA or the elderly was more prevalent in privately insured patients because of a lack of data on individual insurance status. However, data clearly indicate a tendency toward a lower threshold diameter for iAAA repair in fee-for-service countries as a whole rather than in selected centers, suggesting financially biased patient selection.

In the last decade, EVAR has become the treatment of choice for patients with suitable aortic anatomy because of its less invasive nature and lower operative mortality.^9–12^ Anatomic eligibility varies for the currently available devices but is estimated to be 40% to 60% if the device instructions for use are followed.^24^ Small AAAs are known to be more often suitable for EVAR, which may explain the tendency for EVAR in countries with higher rates of small AAA repair.^25^ The use of EVAR for iAAA across countries varied significantly. The reasons for this variation are likely country, center, and surgeon specific. It is notable that the variation in operative technique was equally high between centers within each country. The center-level analysis indicates that although EVAR is centralized to specific centers in some countries such as Denmark and Hungary, it is widely distributed in other countries, and the operative technique used for treatment of patients may be biased by the center where treatment is undertaken.

Data on variations in rAAA repair are especially interesting, given the implications of repairing a small AAA versus one that has met a recommended size threshold. By definition, increasing numbers of small AAA repairs will increase the overall denominator and will decrease the apparent percentage of rAAA in any particular country. This does not necessarily indicate that these repairs prevented rupture, which is difficult to determine without a prospective study. Finland had the highest proportion of rAAA repairs in this cohort. According to a Finnish study, 6.7% of the ruptures occurred in patients in whom the AAA diameter was below the ESVS recommendations for elective repair.^26^ Screening plays a large part in the detection of AAA in some but not most countries and may affect the rate of repair of small AAA. Both the SVS and the ESVS recommend screening in men >65 years of age. Sweden is the only country in the present cohort with a broadly used national AAA screening program, although the US Medicare program offers an initial screening abdominal ultrasound for selected patients. In addition, the rate of accidental detection of AAA may also vary between countries. The potential effect of these factors on AAA repair volume could not be assessed in this study. To assess whether the rate of small AAA repair is dependent on clinical decision making on the threshold for repair or is a potential effect of screening activity, the rate of small AAA was assessed focusing on the iAAA range of 4.5 to 6 cm. This sensitivity analysis did not alter the variation seen between countries (data not shown).

### Limitations

Comorbidities are defined slightly differently in the various registries, and center-specific information is lacking in some countries as a result of pooling of data and patient data protection limitations. However, the international analyses of threshold for iAAA repair, operative technique, and age at repair are not affected by these parameters. The participating registries are validated locally, with several showing excellent internal and external validity for AAA data,^27–30^ but no validation of the ICVR data set as a whole was possible. Some of the participating registries are center or region based and do not cover all AAA repairs for a particular population base, which limits the possibility for detailed epidemiological assessment. ICVR is currently working to harmonize registry data points for future prospective studies, which will be guided by the results of this study. Although this study focused on patient treatment and presentation, additional work is ongoing to determine individual center and country methodology for determining mortality and procedural outcomes to allow more detailed analyses. Despite these limitations, the current ICVR data set allows an effective, broad analysis of the demonstration of international variation in AAA management.

### Conclusions

This study represents a unique international collaboration providing data on the contemporary management of AAA in 11 countries across 3 continents. Despite existing recommendations from professional societies, remarkable differences exist across countries for diameter threshold for iAAA repair, EVAR use, and the management of the elderly. Data suggest that healthcare system and reimbursement may have as much impact on treatment patterns and indication for surgery as scientific studies and guidelines. This study demonstrates the opportunity for further international harmonization of treatment algorithms and additional assessment of the effects of variation on healthcare costs and outcome.

## Sources of Funding

The project and efforts were funded in part by the US Food and Drug Administration through grant 1U01FD005478 (PI Sedrakyan). Views expressed in the publication do not necessarily reflect the official policies of the Department of Health and Human Services; nor does any mention of trade names, commercial practices, or organization imply endorsement by the US government.

## Disclosures

None.

## Supplementary Material

## Clinical Perspective

### What Is New?

- This report by the International Consortium of Vascular Registries is the first such study allowing an assessment of variations in repair of abdominal aortic aneurysms in 11 countries over 3 continents represented by 2 large societies with existing clinical guidelines for abdominal aortic aneurysm management.
- The study demonstrates that significant variation exists between and within countries in the management of both elective and emergent abdominal aortic aneurysm repair with regard to surgical technique and management of elderly patients. These differences in management correlate with healthcare reimbursement models, which may explain some of the variation.

### What Are the Clinical Implications?

- The report indicates that healthcare system and reimbursement have as much impact on treatment patterns and indication for surgery as scientific studies and guidelines, with both positive and negative incentives potentially affecting practice.
- There is an opportunity for further international harmonization of treatment algorithms for abdominal aortic aneurysm repair, especially considering the strength of existing guidelines for intact repair.
