# Supplementary material for: Variations in Abdominal Aortic Aneurysm Care: A Report From the International Consortium of Vascular Registries
Source: Circulation. 2016 Dec 12;134(24):1948–58. doi: 10.1161/CIRCULATIONAHA.116.024870 (PMC5147037; doi:10.1161/CIRCULATIONAHA.116.024870)
Supplement: Supplementary file 1 [file cir-134-1948-s001.pdf]

SUPPLEMENTAL MATERIALS

Supplemental Figure 1. Case selection for analysis.

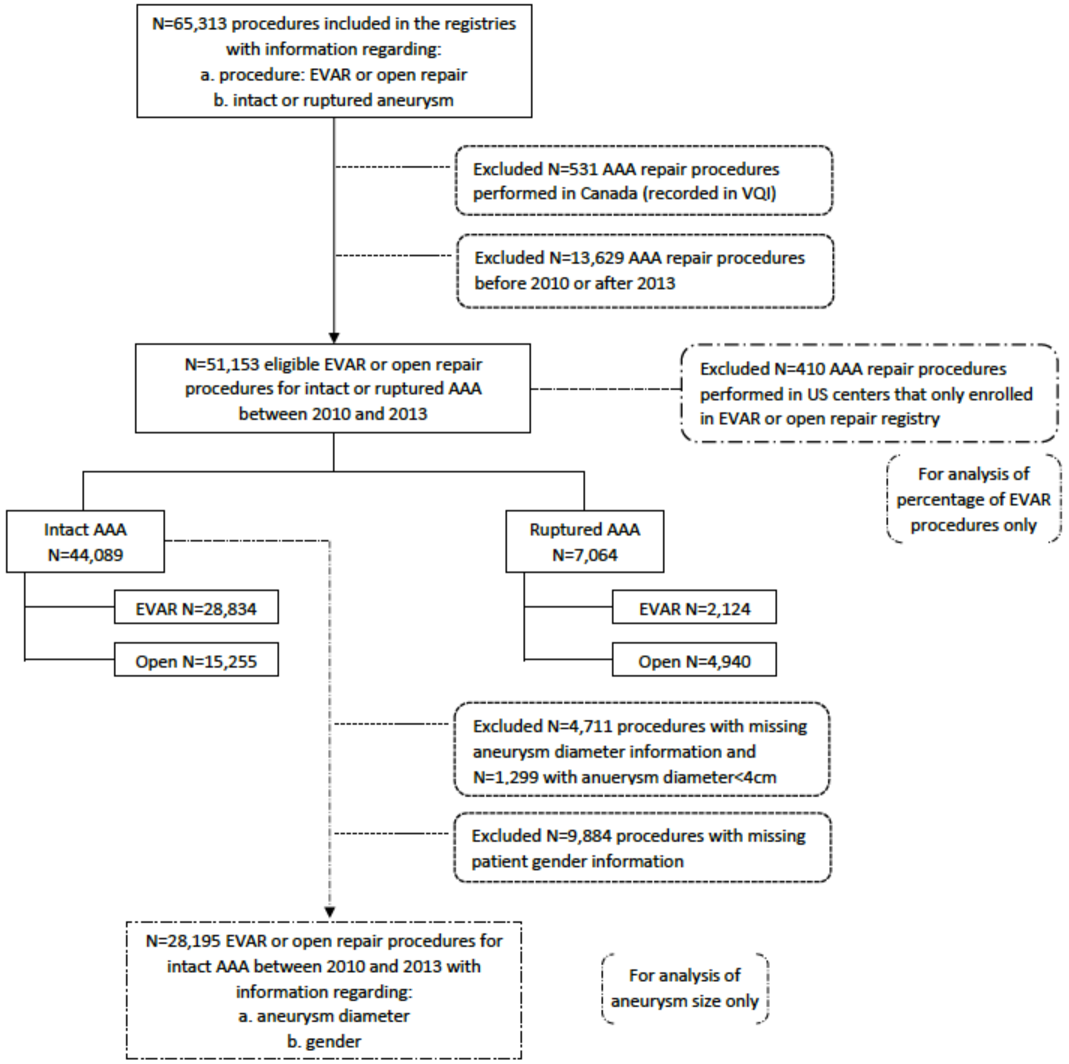

Supplemental Table 1. Number of procedures per year in each registry.

	Intact				Ruptured			
	2010	2011	2012	2013	2010	2011	2012	2013
Australia	1595	1631	1528	1552	363	381	375	325
Denmark	532	596	573	538	212	173	152	211
Finland	119	117	107	118	44	48	57	43
Germany	4144	3935	3320	1173	520	429	382	113
Hungary	221	188	194	246	54	39	53	41
Iceland	11	25	26	14	11	2	4	4
Norway	571	496	529	499	121	89	62	62
New Zealand	230	306	327	351	43	54	71	52
Sweden	1017	1023	923	930	284	246	250	258
Switzerland	521	461	558	634	109	70	86	77
USA	955	2128	3802	5325	100	193	325	476

Supplemental Table 2. Risk factor definitions within countries.

Country	Diabetes	Cardiac history	Pulmonary disease	Stroke history
**Australia**	- Biochemically proven, treated or untreated	- Previous Angina, MI, CABG or PCI<br>- ECG/Stress test	Not collected	Not collected
**Denmark**	- DM(I)<br>- DM(II)	- Angina<br>- Previous myocardial infarction<br>- Previous CABG/PCI	- Treatment for lung disease<br>- Severe dyspnea	- Previous stroke or transient ischemic attack/ amaurosis fugax
**Finland**	- DM(I)<br>- DM(II)<br>- Diet	- Previous MI or CABG, angina pectoris, congestive heart failure, atrial fibrillation	- Diagnosis of chronic obstructive pulmonary disease.	- Previous stroke or transient ischemic attack
**Germany**	- DM(I)<br>- DM(II)	No heart disease vs. stable vs. instable	- Diagnosis of chronic obstructive pulmonary disease.	History of stroke with or without neurological deficite)
**Hungary**	- DM(I)<br>- DM(II)	- Previous MI, PCI or CABG, angina pectoris, congetstive heart failure	- Diagnosis of chronic obstructive pulmonary disease.	Not collected
**Iceland**	- DM(I)<br>- DM(II)	- Previous MI, PCI or CABG<br>- Angina<br>- Congestive heart failure	- Diagnosis of chronic obstructive pulmonary disease.	- Previous ischemic stroke<br>- Previous hemorrhagic stroke
**New Zealand**	- Biochemically proven, treated or untreated	- Previous Angina, MI, CABG or PCI<br>- ECG/Stress test	Not collected	Not collected
**Norway**	- DM(I)<br>- DM(II)<br>- Diet	- Ischemic heart disease<br>- Previous MI or CABG<br>- Congestive heart failure<br>- Valvular heart disease	- Diagnosis of chronic obstructive pulmonary disease.	- Previous stroke or transient ischemic attack
**Sweden**	- DM(I)<br>- DM(II)	- Previous MI, PCI or CABG<br>- Angina<br>- Congestive heart failure	- Diagnosis of chronic obstructive pulmonary disease.	- Previous ischemic stroke<br>- Previous hemorrhagic stroke
**Switzerland**	- DM(I)<br>- DM(II)	- Angina<br>- Previous myocardial infarction<br>- CABG<br>- No cardiac decompensation	- Diagnosis of chronic obstructive pulmonary disease.	Previous stroke
**USA – Vascular Quality Initiative**	- DM(I)<br>- DM(II)<br>- Diet	- Previous MI, PCI or CABG<br>- Angina<br>- Congestive heart failure	- Diagnosis of chronic obstructive pulmonary disease.	Prior CEA or CAS collected in all registries; For Carotid stent and endarterectomy, detailed data regarding the sidedness, location (eye, cortical, vertebrobasilar) and severity are collected, including a pre-op modified Rankin Score

DM(I): Diabetes mellitus type I, DM(II): Diabetes mellitus type II, MI: Myocardial infarction, CABG: Coronary arterial bypass graft,

Supplemental Table 3. Proportion of women undergoing intact AAA repair at a diameter <5.5cm and <5.2cm in different registries.

	<5.5cm, % (95% CI)	<5.2cm, % (95% CI)
Australia	38% (35%-41%)	26% (23%-28%)
Finland	30% (12%-47%)	15% (1%-28%)
Germany	39% (34%-45%)	25% (20%-30%)
Hungary	33% (24%-42%)	23% (15%-30%)
Iceland	15% (0%-35%)	0% (0%-21%)
Norway	40% (28%-40%)	23% (18%-27%)
New Zealand	34% (28%-40%)	17% (13%-23%)
Sweden	34% (30%-38%)	20% (17%-23%)
Switzerland	41% (29%-53%)	23% (13%-34%)
USA	49% (47%-51%)	30% (29%-32%)
